# Transcriptional Changes in Regulatory T Cells From Patients With Autoimmune Polyendocrine Syndrome Type 1 Suggest Functional Impairment of Lipid Metabolism and Gut Homing

**DOI:** 10.3389/fimmu.2021.722860

**Published:** 2021-08-30

**Authors:** Amund Holte Berger, Eirik Bratland, Thea Sjøgren, Marte Heimli, Torgeir Tyssedal, Øyvind Bruserud, Stefan Johansson, Eystein Sverre Husebye, Bergithe Eikeland Oftedal, Anette Susanne Bøe Wolff

**Affiliations:** ^1^Department of Clinical Science, University of Bergen, Bergen, Norway; ^2^ Kristian Gerhard (KG) Jebsen Center for Autoimmune Disorders, University of Bergen, Bergen, Norway; ^3^Department of Medical Genetics, Haukeland University Hospital, Bergen, Norway; ^4^Department of Medicine, Haukeland University Hospital, Bergen, Norway; ^5^Department of Clinical Medicine, University of Oslo, Oslo, Norway; ^6^Department of Anesthesiology and Intensive Care, Haukeland University Hospital, Bergen, Norway

**Keywords:** APS-1, autoimmune polyendocrine syndrome type 1, Tregs (regulatory T cells), RNAseq analysis, transcriptomics, flow cytometry, autoimmune disease

## Abstract

Autoimmune polyendocrine syndrome type I (APS-1) is a monogenic model disorder of organ-specific autoimmunity caused by mutations in the *Autoimmune regulator (AIRE)* gene. AIRE facilitates the expression of organ-specific transcripts in the thymus, which is essential for efficient removal of dangerous self-reacting T cells and for inducing regulatory T cells (Tregs). Although reduced numbers and function of Tregs have been reported in APS-I patients, the impact of AIRE deficiency on gene expression in these cells is unknown. Here, we report for the first time on global transcriptional patterns of isolated Tregs from APS-1 patients compared to healthy subjects. Overall, we found few differences between the groups, although deviant expression was observed for the genes *TMEM39B, SKIDA1, TLN2, GPR15, FASN, BCAR1, HLA-DQA1, HLA-DQB1, HLA-DRA, GPSM3 and AKR1C3.* Of significant interest, the consistent downregulation of *GPR15* may indicate failure of Treg gut homing which could be of relevance for the gastrointestinal manifestations commonly seen in APS-1. Upregulated *FASN* expression in APS-1 Tregs points to increased metabolic activity suggesting a putative link to faulty Treg function. Functional studies are needed to determine the significance of these findings for the immunopathogenesis of APS-1 and for Treg immunobiology in general.

## Introduction

Immunological homeostasis and maintenance of health rely on a complex network of structured processes. These are organized in several layers to ensure that the immune system will efficiently stop invaders without harming the host. T cells are central in initiating and maintaining specific immune responses. T cells with suppressive capacity, i.e. regulatory T cells (Tregs), constitute about 5-7% of the CD4^+^ T cell repertoire, and have a critical role in maintaining homeostasis by dampening inexpedient immune responses in the peripheral blood and tissues (1, 2). They are characterized by expression of the linage-defining transcription factor forkhead box P3 (FOXP3), and are equipped with a distinct epigenomic signature, especially at the *FOXP3* locus (1–3).

The primary location for development and maturation of Tregs is in the thymus through positive and negative selection in response to self-antigen stimulation (thymic or natural Tregs). Lately, it has become apparent that Tregs are also generated outside the thymus (peripheral Tregs or pTregs) and, at least in mice, can be converted from Cd4^+^Cd62l^+^ central memory T cells in blood (4). Mature Tregs are present in peripheral blood, tissues and especially in draining lymph nodes, where they control antigen presentation. They may also recirculate back into the adult thymus in order to control thymic output of *de novo* Tregs (5). Another important aspect of Tregs is their ability to mimic phenotypes of other T cells, e.g. Th1 and Th17 through expression of the relevant signature transcription factors (T-bet and RORγt, respectively) (6, 7), and modulate the environment with their cytokine profile. This has challenged the previous view that the CD4^+^ cells are static with either a T effector cell or a Treg phenotype.

Autoimmune polyendocrine syndrome type I (APS-1) is a severe monogenic autoimmune disorder characterized by autoimmune manifestations in endocrine glands and ectodermal tissues. Mutations in the *Autoimmune Regulator (AIRE)* gene (8–10) cause impaired immunological tolerance. Interestingly, AIRE is involved in all layers of tolerance induction: It orchestrates the expression of tissue-specific antigens in the thymus to be presented to developing T cells during negative selection (11) and has a further distinct role in the generation of Tregs (12). Aire specifically induces fetal generation of distinct subtypes of Cd4^+^FoxP3^+^ nTregs in mice (13), is involved in Ccr6-dependent recirculation of n-/p-Tregs into the thymus (5), and in generating pTregs in the periphery (14). We and others have previously found either decreased frequencies and/or impaired function of Tregs in patients with APS-1 (15–17), however no in-depth studies of the Tregs transcriptomes in these patients have been conducted. Correct function of Tregs is essential for a healthy immune system, exemplified by FoxP3 deficient mice and patients with *FOXP3* mutations, both suffering from numerous autoimmune manifestations (3, 18). Also, single nucleotide polymorphisms (SNPs) linked to common autoimmune diseases are enriched in DNA regions of CpG demethylation crucial for Treg cell development and function (19).

With an increase in available immune therapies aiming to utilize the specific capacity and plasticity of Tregs to downregulate immune responses, it is crucial to understand suppressive and counter-acting activities of Tregs, and their contribution to autoimmune disease.

In order to investigate whether Treg transcriptional activity and not just selection is affected by AIRE dysfunction, we have undertaken a descriptive study of isolated Tregs from APS-I patients and compared both protein expression and transcriptomes to healthy controls. We hypothesize that transcriptome analyses of purified Tregs in APS-1 patients, with their dual failures of central and peripheral tolerance, could shed light on the pleiotropic nature of AIRE in Tregs and immune function in general.

## Methods

### Patients and Controls

Nine patients with APS-1 (confirmed with *AIRE* mutational analysis and autoantibody screening against IFN-ω) [44% males, mean age 49.2 years (range 30-63)] and 21 healthy subjects were included in this study (**Table 1**). The patients have been reported previously (20). Seventeen of the healthy controls were included in the RNA sequencing of Tregs (HC1-17), 59% males, mean age 49.2 years (range 20-76), while eight healthy subjects were included in the flow cytometry analyses of Tregs (HC1-4+18-21), 87% males, mean age 44.4 years (range 25-66).

**Table 1:** Demographics of included APS-1 patients.

ID	Sex	Age (incl.)	AIRE mutations	Manifestations^#^	Autoantibodies^¤^	Classification^1^
P1 (+flow)	F	60	c.976-979del13/del exon 1-8	AAD, HP, C, G, E, A, N	21OH, SCC, TPH, NALP5, 17OH, IL22, IFNw	del13
P2 (+flow)	F	45	c.879+1G>A/c.879+1G>A	AAD, C, E	21OH, NALP5, 17OH, IFNw	splice
P3 (+flow)	M	60	c.879+1G>A/c.879+1G>A	HP, T1D, V, T, E, C	AADC, GAD, TPH,NALP5, 17OH, 21OH, TH, IFNw	splice
P4 (+flow)	M	49	c.976-979del13/c.976-979del13	AAD, C	21OH, SCC, GAD, IL22, IFNw	del13
P5	M	55	c.769C>T/c.1249dupC	E, C	AADC, IFNw	R257X
P6	M	34	c.22C>T/c.967_979del13	AAD, HP, C, M, E	21OH, AADC, IL17, IL22, -IFNw	del13
P7	F	63	c.1336T>G/c.976-979del13	AAD, E	21OH, 17OH, SCC, GAD, IFNw	del13
P8	F	30	c.976-979del13/c.976-979del13	HP, E, T, M	NALP5, IFNw	del13
P9 (+flow)	F	47	c.934G>A(dominant)	HP, E, G, B12, M	NALP5, IFNw	C311Y

P, Patient; HC, Healthy control; F, Female; M, Male; NK, not known; (+flow) means that they were included both in the transcriptional study and in the flow cytometry approach.

^#^Manifestations: AAD, Autoimmune adrenocortical insufficiency; HP, Hypoparathyroidism; C, Chronic mucocutaneous candidiasis; G, Gonadal failure; E, Enamel dysplasia; A, Asplenia; N, Nail pitting; T1D, Type I diabetes; V, Vitiligo; T, Thyroid disease; M, Malabsorption (gastrointestinal dysfunction); B12, B12 deficiency.

^¤^Autoantibodies: 21OH, 21-hydroxylase; SCC, side-chain cleavage enzyme; TPH, tryptophan hydroxylase; NALP5, NACHT, LRR and PYD domains-containing protein 5; 17OH, 17-hydroxylase; IFN-ω, interferon omega; IL22, interleukin-22; GAD, glutamic acid decarboxylase; AADC, aromatic L-amino acid decarboxylase.

^1^This classification of mutations is used in **Figures 2**, **3** and **4**.

### Flow Cytometry Analysis

The Treg flow cytometry panel was inspired by a clinical Tregs workshop (21). Blood was sampled in heparin-containing tubes and peripheral mononuclear cells (PBMCs) were isolated by Ficoll density gradient separation following standard procedures before freezing down at -150°C in AB serum with 10% DMSO. For flow analysis, thawed PBMCs from 5 patients and 8 controls (see **Table 1**) were added two microliter of Fc block for 15 min before addition of the extracellular antibodies anti-CTLA4 BV421 (BN13, Biolegend, San Diego, CA), anti-CD39 PE (ebioA1, Invitrogen, Carlsbad, California, USA) and anti-CD3 V500 (UCHT1); anti-CD4 Alexa Fluor 700 (RPA-T4); anti-CD8 PerCP-Cy5.5 (SKI); anti-CD25 PE-Cy7 (2A3); anti-CD45RA APC-H7 (HI100) and anti-CD31 BV650 (L133.1) from BD (Franklin Lakes, New Jersey, USA). The cells were resuspended in 1 mL PBS and live/dead Fixable Yellow Dead Cell stain kit (Invitrogen) was added in a 1:1000 dilution. Staining of intracellular markers [anti-FOXP3 PE-CF594 (259D/C7, BD), anti-HELIOS APC (22F6, Biolegend), and anti-Ki-67 (20Raj1, Invitrogen)] was subsequently performed using the eBioscience Anti-human FOXP3 Staining Set as explained by the manufacturer with the change that the cells were fixed overnight. Acquisition was executed by a LSR Fortessa flow cytometer and data was analyzed by FlowJo version 10.4.

### Isolation of Tregs and Purification of RNA

Fresh blood samples (15-25 ml) were collected in EDTA tubes and Tregs were enriched using the MacsExpress Treg isolation kit according to the manufacturer’s protocol (Miltenyi Biotech, Bergisch Gladbach, Germany), resulting in samples constituting 43-77% Tregs (mean controls 65%; mean patients 60%), validated by flow cytometry (CD4^+^CD25^hi^CD127^-^). Positively selected cells were prepared for flow cytometry sorting (FACS) by staining with antibodies obtained from Miltenyi Biotech: anti-CD4 FITC (M-T466), anti-CD25 PE (4E3) and anti-CD127 APC (cMB15-18C9). After 30 min incubation in the dark at 4°C, the cells were washed by addition of 2 ml rinsing buffer (Miltenyi Biotech) and centrifugation for 10 min at 350g at 4°C. The resulting pellet was resuspended in PBS with 0.5% BSA and CD4^+^CD25^hi^CD127^-^ cells (Tregs) were sorted into aliquots of 5000 cells by the use of BD FACSAria SORP or FACSymphony S6 directly into the buffer containing 75 μl RLT plus 10% v/v β-mercaptoethanol. RNA from the lysate was immediately purified using the RNeasy Micro Plus Kit, following the recommendations from the supplier (Qiagen, Venlo, Netherlands). The RNA quality was assessed by the Agilent Bioanalyzer, using the Agilent 6000 Pico kit or by TapeStation with the High Sensitivity D5000 ScreenTape kit (Agilent, Santa Clara, CA, USA). Concentrations were measured by Qubit RNA HS Assay kit (Invitrogen). Samples were stored at -80°C until use.

### RNA Sequencing

cDNA was made from RNA and amplified using the SMART-Seq v4 Ultra Low Input RNA Kit according to the manufacturer’s protocol (Takara Bio, Madison, Wisconsin, USA). Undiluted RNA samples from the 5000 cells aliquots were used as input. cDNA amplification followed a program of 10 (for samples P1-P4 and HC1-HC4) and 14 (for samples P5-P9, R1 and HC 5-17) cycles (see **Table 1** for subject information). The cDNA was purified by the Agencourt AMPure XP Kit (Beckman Coulter, Pasadena, CA, USA) and assessed by an Agilent Bioanalyzer (Agilent High Sensitivity DNA Kit) or TapeStation (High Sensitivity D5000 ScreenTape kit) according to the manufacturer’s protocols (Agilent). The samples were then stored at -20°C before use.

Library preparation was performed using the Nextera XT DNA Library Preparation kit and the Nextera XT Index kit according to the manufacturer’s protocol (Illumina, San Diego, CA, USA). The input cDNA was diluted according to the results from the Agilent Bioanalyzer/TapeStation and Qubit™ dsDNA HS Assay Kit (concentrations between 11-63 ng/microliter and addition of 3.5 to 5 microliter for library preparations). Unique combinations of i5 (5 μl) and i7 (5 μl) index adapters were added to each respective sample. Constructed libraries were then assessed with the High sensitivity D5000 ScreenTape kit, by TapeStation (Agilent). The final libraries consisted of fragments mostly between 250-2500 bp, with a peak of 500-1000. Sequencing of the products was performed by the Bergen Genomics core facility, using a HiSeq4000 sequencer according to the Illumina TruSeq Stranded mRNA protocol.

### Bioinformatics Analysis of RNA Sequencing Data

Quality control of the sequencing data was performed using the FastQC software (v.0.11.9) (22), and reads were aligned to the GRCh38.p12 (build 100) reference transcriptome by the Kallisto pseudoaligner [v 0.46.2 (23)] in quant pair-end mode using a k-mer length of 31. The pseudoalignments were subsequently aggregated to the genome using the tximport (v.1.16.1) (24) package in R (v.4.0.2) and annotated using biomaRt (v.2.44.4) (25). Differential expression analysis was performed using the DESeq2 package (v.1.28.1) (26) using the default significant level of 10% FDR. In order to maximize power in this analysis the Independent Hypothesis Weighting (IHW R package) (27) multiple testing method was used to control for false discovery rate, and the Approximate posterior estimation for GLM coefficients (apeglm R package) (28) method for log2 fold change shrinkage was used to remove noise. BAM files for gene visualization were generated using STAR aligner (v. 2.7.5a) (29) and indexed using Sambamba (v. 0.8.0) (30).

### Statistical Analysis and Plot Generation

All datasets were imported into RStudio [v. 1.3.1073 (31)], and were treated using tidy data principles with the tidyverse R packages (v. 1.3.1) (32). Tidyverse package ggplot2 was used to visualize the data in various volcano plots, violin plots, dot plots, box plots, and heatmaps. The cowplot package (v. 1.1.1) was utilized to combine different ggplots, tidyverse package scales was used to make sensible axis scales, ggrepel (v. 0.9.1) was used to annotate plots without overlapping labels, and ggpubr (v. 0.4.0) was used to make some publication ready plots. Colorblind friendly color scales were generated using RColorBrewer (v. 1.1-2) and Viridis [v. 0.6.0 (33)]. A non-parametric, two-tailed Mann-Whitney *U* test by the rstatix package (v. 0.7.0) was applied to analyze the differences between patients and controls regarding the Tregs subpopulations in flow cytometry, and results were adjusted for multiple testing using Benjamini-Hochberg FDR correction. Results were found statistically significant if FDR < 0.05. Two-sided Mann-whitney U tests were also performed on the age distribution data of the sequenced control and patient groups, with a significance threshold level of P <= 0.05.

Gene visualizations of reads were performed using the Gviz R package [v. 1.34.1 (34)] where EnsDb.Hsapiens.v100 were used as the reference genome supplied by the AnnotationHub R package (v. 2.22.1).

### Pathway Analysis

String (https://string-db.org/cgi/network) was utilized to perform pathway analysis for the differentially expressed genes identified. This web tool can mediate identification of affected pathways by finding associations between selected proteins based on text-mining, co-mentions in PubMed abstracts, known interactions (experimentally determined or from curated databases) or predicted interactions.

### Ethics

This project was conducted in compliance with the Declaration of Helsinki and approved by the Regional Ethics Committee of Western Norway (approval numbers 2009/2555 and 2018/1417). All patients were recruited from the Registry for organ specific autoimmune disorders (ROAS), Haukeland University Hospital, Norway, and gave written informed consent for participation. Samples from healthy subjects were obtained from the Haukeland University Hospital blood bank. Data was stored and analyzed on secure servers throughout the whole project (SAFE, University of Bergen; TSD, University of Oslo and HUNT Cloud, Norwegian University of Science and Technology, all Norway).

## Results

### Flow Cytometry Profiling of Tregs From APS-1 Patients

**Figure 1:**
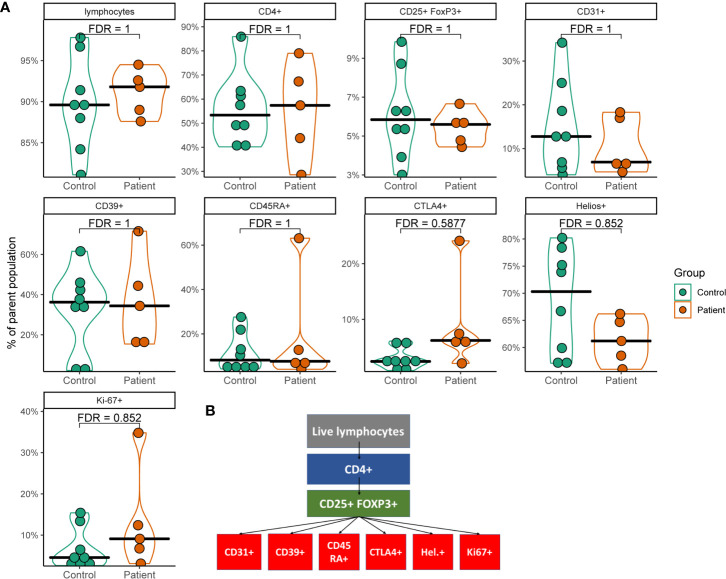
Violin plots of flow cytometry data from a subset of APS-1 patients and healthy subjects. **(A)** Plots show different cell populations positive for markers and their percentages relative to the gated parent population. Statistical analysis was performed by using Mann-Whitney U test with Benjamini-Hochberg correction (FDR) in R. Gating strategy is displayed in **(B)**.

In order to investigate the impact of *AIRE* mutations on Treg distribution and protein expression within Tregs, PBMCs from five of the nine included APS-1 patients and eight healthy subjects were profiled for common Tregs surface markers using flow cytometry (**Figure 1A**). Tregs were identified by the markers CD4^+^CD25^hi^FOXP3^+^, and subtypes hinting at functional properties were then assessed within this cohort of cells according to the gating strategy outlined in **Figure 1B** and shown in **Supplementary Figure 1**. There were no major differences between patients and controls in the frequency of CD4^+^ cells within CD3^+^ PBMCs, nor in Tregs within the CD4^+^ parent cohort [Tregs mean patients 5.5% (range 4.4-6.7%) *versus* mean controls 6.1% (range 3.0-9.9%)]. Furthermore, there were no notable variation in the thymic recent emigrant/epithelial marker CD31, the ectoenzyme for functional capacity of Tregs CD39, the naïve marker CD45RA, nor the activation marker Ki67 although one outlier in each of CD45RA and Ki-67 in the patient group exhibited increased expression compared to controls. An overall trend towards lower amounts of HELIOS-expression and higher levels of CTLA-4- signals could be observed within the APS-1 cohort, but these observations did not reach statistical significance after multiple testing corrections (**Figure 1A**).

### Sample Characterization and Quality Controls of the cDNA Libraries Made From Tregs-RNA

Tregs were isolated from whole blood by magnetic beads and then FACS from a total of 9 APS-1 patients and 17 age and sex matched healthy subjects (**Supplementary Figure 2A**). The largest group of patients (n=5) shared the 13bp-deletion mutation in *AIRE*. Although two of these were homozygous for c.976-979del13, P1 had this mutation together with a large deletion spanning at least exon 1 to intron 8 (35) while two were compound heterozygous for the 13 bp deletion together with two different missense mutations (classified in **Table 1**). There were no differences in age or sex distribution between patients and controls (**Supplementary Figures 2B, C**).

Because of limitations regarding the collection of Tregs from APS-1 patients, the sample collection, library preparation and sequencing were performed in two batches, visualized in **Supplementary Figure 2D**. There were no statistical differences in the age distribution nor genders within each batch between patients and controls (**Supplementary Figures 2E, F**).

Five-thousand cells were FACS sorted from up-concentrated Tregs of each patient and control, RNA was isolated and then sequenced. The first batch was sequenced to an average read depth of 70.3 million bases, while the second batch was sequenced to an average read depth of 42 million bases (**Supplementary Figure 3**).

Principal component analysis (PCA) plots were compiled for the sequencing data with the two largest PCs explaining 17% and 12% of the variance, respectively. The metadata variable closest to explaining this variance was batch 1 or 2, indicating a possible issue with batch effects (**Supplementary Figure 4**). Hence, batch-variance were included as a covariate in the differential expression model design to analyze gene expression in Tregs from APS-1 patients compared to controls. However, as a quality control measure, two individuals were sampled in both sequencing batches representing two different timepoints for Treg-isolation. PCA plots of these patients displayed very little difference between batches, indicating that the impact of any batch effect may be small. We included only the initial samples for downstream analyses.

### Treg Characterization by Transcriptomic Profiling

**Figure 2:**
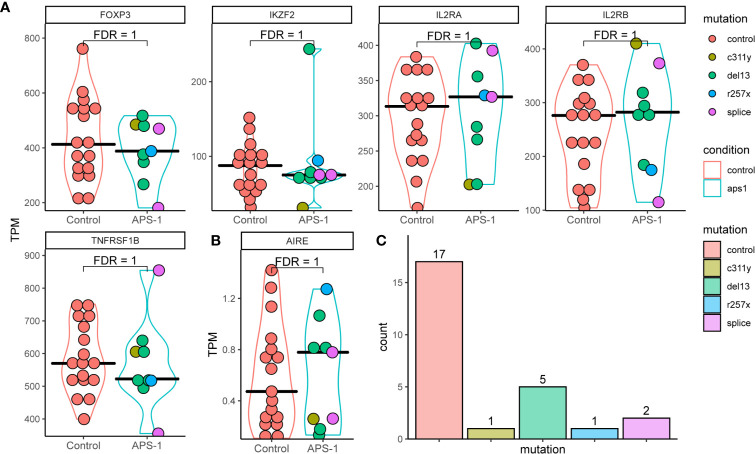
Violin plots of RNA expression levels in transcripts per million (TPM) of important regulatory T cell markers in Tregs of APS-1 patients (cyan) and healthy subjects (red). **(A)** APS-1 mutations are indicated by the color of the dots within the violin plot. AIRE expression in TPM for the different groups shown in **(B)** Histogram of distribution of patients and controls according to genotype in **(C)** FDR values taken from differential expression analysis using DESeq2 in R.

Successful Treg isolation and sequencing was verified by exploring the gene expression of five genes characteristic of Tregs (36). Interrogating the gene expression of the master regulator of the Treg pathway *FOXP3*, HELIOS (*IKZF2*), Interleukin-2 receptor alpha and beta chains (*IL2RA* and *IL2RB*), as well as TNF receptor superfamily member 1B (*TNFRSF1B* also known as CD120b) showed relatively high expression in transcripts per million (TPM) (**Figure 2A**). They were further expressed at similar levels with no significant difference in distribution between patients and control. This expression signature confirmed that the isolated cells were Tregs, and that there is little difference in these signature Treg genes between the healthy controls and APS-1 patients within the dataset. Analyzing whether the distribution of reads conforms with the exons within the gene or whether these reads may be misaligned can reveal false differential gene expression findings caused by low expression levels. In this case the RNA sequencing read distributions across these genes showed stable exon expression (data not shown).

As the *AIRE* gene, which is impaired in APS-1 patients, is implicated in Tregs development and function (14–17, 37, 38), we performed a detailed analysis of its’ expression. The results show low level (sub 2 TPM) *AIRE* expression in most patients and controls (evenly distributed between the different mutations). However, there were no significant difference in gene expression between the groups, and no consistent difference in expression of *AIRE* or the 5 genes characteristic of Tregs between the *AIRE* mutations encountered in this dataset **(** **Figures 2B, C**). While expression levels were relatively low, read distribution conformed mainly to within exons in *AIRE*, confirming *AIRE* mRNA expression.

### Differential Expression of Genes in APS-1 Patients *Versus* Controls

**Figure 3:**
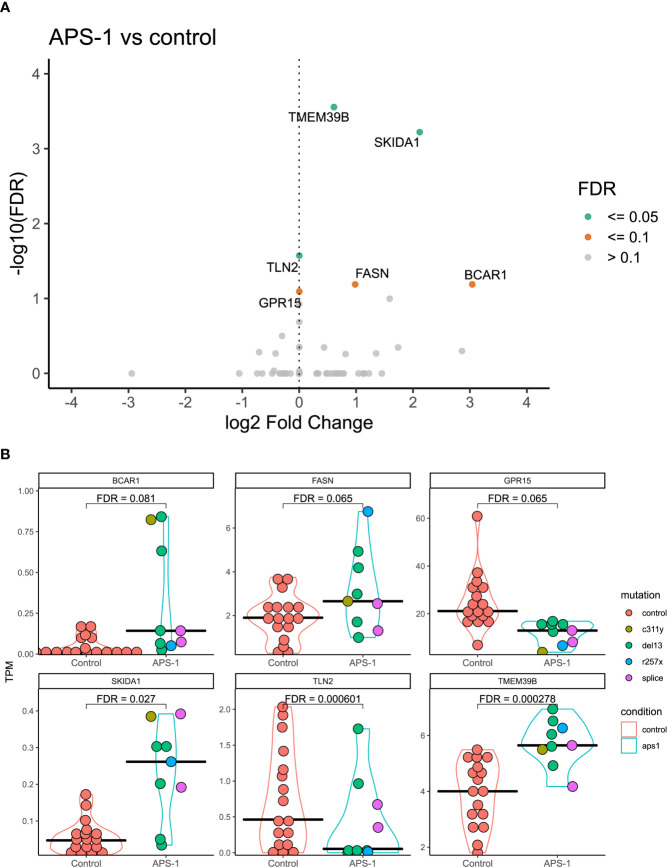
Differential expression analysis of regulatory T cells from APS-1 patients and healthy subjects. **(A)**. Volcano plot of the most significantly differentially expressed genes with the magnitude of differential expression in log2 fold change and the significance level in -log10 FDR. **(B)** Violin plots showing the absolute expression level in transcripts per million (TPM) in all samples for the genes identified as significantly differentially expressed at a 10% FDR level in **(A)**. FDR values on violin plots taken from differential expression analysis using DESeq2 (v. 1.30.1)in R (v. 4.0.2.).

Differential expression analysis was performed using DESeq2, taking advantage of an experimental design consisting of batch and condition, where condition indicated APS-1 or control and batch was treated as a covariate in the statistical modelling. Overall, the results indicated very small differences between APS-1 patients and healthy subjects as most genes were not differentially expressed (**Tables 2A**, **B** and **Figure 3A**). The most differentially expressed genes were *Transmembrane protein 39 (TMEM39B), Ski/Dach domain-containing protein 1 (SKIDA1)*, and *Talin 2 (TLN2)*, expressed at a 5% FDR level at log2 fold changes of 0.61, 2.12, and -3.82e-06 respectively. Using a less stringent 10% FDR level, the genes *G Protein-Coupled Receptor 15 (GPR15), Fatty Acid Synthase (FASN)*, and *Cas Scaffolding Protein Family Member 1 (BCAR1)* were also found to be differentially expressed, with log2 fold changes of -1.46e-05, 0.98, and 3.03. Of these, *SKIDA1*, *BCAR1* and *TLN2* were lowly expressed at below 2 TPM (**Figure 3B**). Gene visualization of read distribution using the Gviz r package shows reads aligning to exons in *BCAR1*, *FASN*, *GPAR15* and *TMEM39B*, while reads in *SKIDA1* and *TLN2* seem more spurious (**Supplementary Figure 5**).

**Table 2A:** Gene expression significantly up or down-regulated in APS-1 patients’ Tregs’ compared with healthy subjects (Adjusted p<0.1).

Gene name	ENSG	baseMean	log2FoldChange	IfcSE	p-value	FDR
TMEM39B	ENSG00000121775	194.050369	0.608219	0.118560	1.192890e - 08	0.000278
GPR15	ENSG00000154165	1897.999700	(-)1.463056e - 05	0.001442	1.597193e - 05	0.080631
FASN	ENSG00000169710	253.271931	0.982477	0.288854	1.069005e - 05	0.064760
TLN2	ENSG00000171914	23.769834	(-)3.818718e - 06	0.001442	3.434099e - 06	0.026692
SKIDA1	ENSG00000180592	16.385416	2.115451	0.448062	5.152753e - 08	0.000600
BCAR1	ENSG00000285460	8.758959	3.038393	0.797175	9.522990e - 06	0.064760

All patients (N=9) vs. all controls (N=17).

**Table 2B:** Gene expression significantly up or down-regulated in APS-1 patients’ Tregs’ compared with healthy controls (FDR<0.1).

Gene name	ENSG	baseMean	log2FoldChange	IfcSE	p-value	FDR
TMEM39B	ENSG00000121775	194.050369	0.558467	0.159874	1.854040e - 05	0.070926
AKR1C3	ENSG00000196139	504.008087	(-)6.907416e - 06	0.001442	7.871471e - 05	0.068547
HLA-DRA	ENSG00000206308	594.779432	(-)1.207145e - 06	0.001442	7.612310e - 08	0.00046
HLA-DRA	ENSG00000230726	594.779432	(-)1.207145e - 06	0.001442	7.612310e - 08	0.000225
HLA-DQB1	ENSG00000231939	744.702414	(-)5.261466e - 07	0.001442	3.742834e - 06	0.007379
HLA-DQA1	ENSG00000232062	285.978473	(-)2.139251e - 07	0.001442	6.002144e - 26	1.829393e-21
GPSM3	ENSG00000233490	32.823953	(-)1.902073e - 07	0.001442	4.526263e - 22	6.507526e-18
HLA-DRA	ENSG00000234794	594.779432	(-)1.207145e - 06	0.001442	7.612310e - 08	0.000154

Patients with the c.976-979del13 (N=5) vs all controls (N=17).

**Figure 4:**
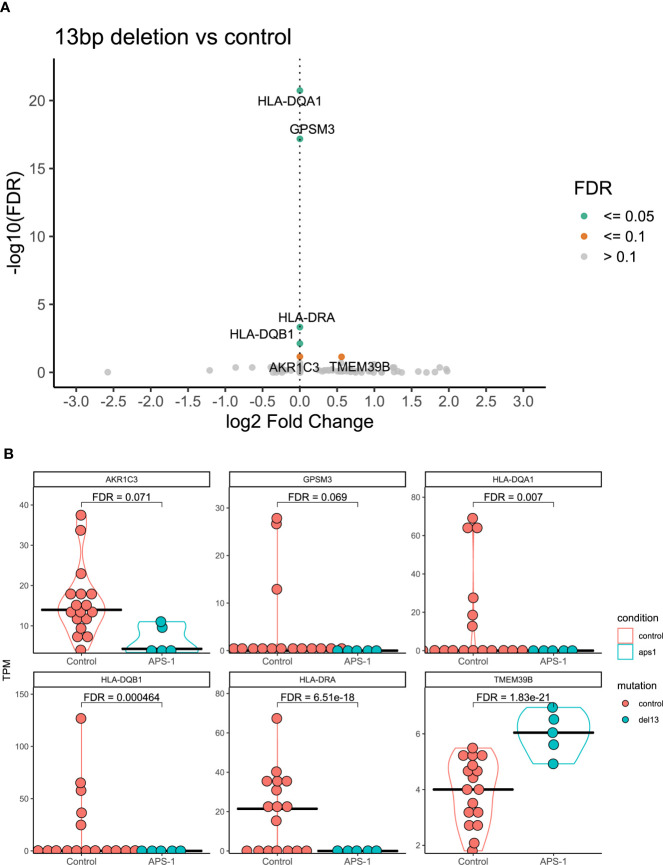
Differential expression analysis of regulatory T cells from APS-1 patients with the 13bp deletion and controls. **(A)** Volcano plot of the most significantly differentially expressed genes with the magnitude of differential expression in log2 fold change and the significance level in -log10 FDR. **(B)** Violin plots showing the absolute expression level in transcripts per million (TPM) in all samples for the genes identified as significantly differentially expressed at a 10% FDR level in **(A)** FDR values on violin plots taken from differential expression analysis using DESeq2 in R.

Because clinical and experimental findings suggest that different *AIRE* mutations have different impact on AIRE function and activity (e.g. from complete loss of function to residual activity) (39, 40), differential expression analysis was also performed using only the subset of APS-1 patients (n=5) with the 13bp deletion mutation, the most common mutation in the dataset. Results of this subgroup analysis showed that the MHC-II complex alleles *HLA-DQA1, HLA-DQB1, HLA-DRA*, and *G Protein Signaling Modulator 3 (GPSM3)* were significantly differentially expressed at a 5% FDR significance level between patients with the 13bp deletion and healthy controls (**Figure 4A**), with log2 fold changes of -2.14e-07, -5.26e-07, -1.21e-06, and -1.90e-07 respectively. Reducing the significance level to 10% FDR, *TMEM39B* again appeared significant in this subgroup and *Aldo-Keto Reductase Family 1 Member C (AKR1C3)* showed differential expression with log2 fold changes of 0.56 and -6.91e-06. Notably, for *GPSM3* and the *HLA-DR* and *HLA-DQ* hits, this might be driven by high expression in a few of the controls (**Figure 4B**).

To investigate whether subtle changes in selected Treg related genes could be missed by a stringent per gene significant level as reported by Ferraro et al. (41), volcano plots were generated on the subset of genes reported as “193 genes that are most differentially expressed between Tregs and effector T cells”. However, none of these genes were found differentially expressed at a sub significant level in the dataset when comparing Tregs from APS-1 patients to healthy controls (data not shown).

### Pathway Analyses

Analysis using String was performed to investigate whether any functional link could be identified between the differentially expressed genes in this study, and additional well-established Treg associated genes (**Supplementary Figure 6**). The five highly relevant Tregs-genes *FOXP3*, *IKZF2*, *IL2RA*, *IL2RB*, and *TRNRSF1B* were included in the model. Furthermore, String gene ontology enrichment analysis was performed in order to investigate any links between gene ontology and biological processes for the differentially expressed genes (**Supplementary Table 1**). As antigen presenting cells and their HLA-proteins which present peptides to T cells have pivotal roles in immune reactivity and tolerance, it is to no surprise that String reported an association between FOXP3 and HLA-DQB1 as “being co-mentioned” in PubMed-abstracts in numerous articles, e.g (42). The gene ontology enrichment strengthened this association by pointing out the relevance of HLA-molecules for proper immune function. String further highlighted a link between SKIDA1 and IKZF2, based on experimental evidence and text-mining on interaction of putative homologues of these proteins in other organisms (*C. elegans* and *D. melanogaster*). TLN2 and BCAR1 were suggested to occasionally be co-expressed in homo sapiens, which could imply functional associations in one or several of the following functional pathways; membrane “ruffle” as part of cell motility, Rap1-signalling and/or in cellular focal adhesion.

## Discussion

We have shown, for the first time, that transcriptional changes within Tregs of APS-1 patients could imply impairment in Treg lipid metabolism and gut homing, respectively. Although our flow cytometry and transcriptomic data show little overall difference between APS-1 patients and healthy controls, some important identified deteriorations could interfere with proper Treg function. For the flow cytometry profiling, there were some trends among the APS-1 patients of increased frequencies of the inhibitory receptor CTLA-4, and decreased frequencies of HELIOS and CD31 expressing cells. However, with few patients the data is difficult to interpret. These findings could however corroborate previously suggested impairment of thymic generation of Tregs in APS-1 (15, 37).

Using differential expression analysis based on RNAseq data, we found that the six genes *GPR15, TMEM39B, FASN, TLN2, SKIDA1* and *BCAR1* differ significantly in their expression level between patients with APS-1 and controls. Although this low number of differentially expressed genes supports that there are no extensive impairments of Tregs in APS-1 patients, several of the identified putative dysregulated Tregs-genes have important functional implications for Tregs.

One of our more interesting findings concerns *GPR15*, encoding a G protein-coupled receptor that acts as a homing receptor for the gut and the lamina propria (43). The protein is regulated by the Ahr-RORγt-FOXP3 axis (44) and has been found to be upregulated in immune cells in blood and affected tissues in several autoimmune disorders; thus it could be important in modulating inflammation (45–48). Interestingly, the gut-homing property seems to include FOXP3^+^ Tregs in particular, indicating that GPR15 plays a role in mucosal immune tolerance largely by regulating the influx of Tregs. Recently, it was demonstrated that patients with colorectal cancer have increased frequencies of GPR15^+^ Tregs in peripheral blood, correlating with high numbers of GPR15^+^ Tregs infiltrating the colonic cancerous lesions (49). These observations indicate that GPR15 directs Tregs into the colon, thereby modifying the tumor microenvironment and promoting intestinal tumorigenesis. Considering this phenomenon, it is important to bear in mind that a substantial fraction of APS-1 patients have gastrointestinal manifestations and highly dysregulated immune responses against intestinal commensal microbiota (50, 51). The latter has also been shown to be associated with Tregs dysfunction. In addition, gut biopsies of APS-1 patients have revealed reduced numbers of FOXP3+ cells in these tissues compared to healthy controls (51). It may be possible that the consistently downregulated GPR15 expression in APS-1 patients’ Tregs could reflect an opposite pattern than colorectal cancer: Lower expression of GPR15 in peripheral blood Tregs, alternatively lower numbers of GPR15^+^ Tregs in general, and consequently lower numbers of Tregs in the gastrointestinal compartments potentially unleashing a proinflammatory and autoimmunity-prone microenvironment. GPR15^+^ Tregs may also be indirectly linked to AIRE deficiency as tumor associated GPR15^+^ Tregs in colorectal cancer has been shown to have a thymic origin (49).

The most statistically significant finding in this study was the identification of higher expression of *TMEM39B* in APS-1 patients. Both TMEM39B and its paralogue TMEM39A has been shown to be important for autophagy, possibly by controlling the spatial localization and synthesis of phosphatidylinositol 4-phosphate (52). Although autophagy mediates Tregs lineage stability and survival fitness (53), we cannot confirm any connection between TMEM39B, Tregs and autophagy. TMEM39B is, according to the Human Protein Atlas, expressed in many tissues, but particularly in the thymus and lymphoid tissues. It has also been shown to be overexpressed in lymphoid malignancies, indicating involvement in lymphocytic cellular growth and survival (54). Additional studies are needed to investigate the consequence of increased TMEM39B expression, and autophagy in APS-1 patients.

*FASN* is another differentially expressed gene which showed upregulation in Tregs from APS-1 patients compared to healthy controls. This enzyme is important in fatty acid synthesis and metabolism by catalyzing the condensation of acetyl-CoA and malonyl-CoA to generate longer acyl chains, which can subsequently be used as a precursor to generate fatty acids and phospholipids. Of particular relevance for adaptive immunity is the requirement of metabolic reprogramming in T cell activation, and fatty acid metabolism involvement in controlling the balance between an effector and a regulatory phenotype (55). Tregs require more active glycolysis, mitochondrial respiration, and fatty acid oxidation, compared to effector T cells indicated by elevated expression levels of *FASN* amongst other genes encoding metabolic enzymes (56). Specifically, it has been found that *de novo* fatty-acid synthesis mediated by FASN determines Treg maturation and if FASN is deleted from Tregs, tumor growth is inhibited (57). Indeed*, in vivo* inhibition of FASN in Th17 cells leads to reduction of autoimmune disease in mice (58), perhaps reflecting the crucial balance between Tregs and Th17 in preventing autoimmunity. We found an upregulation of *FASN*, indicating that Tregs of APS-1 patients may have more active fatty acid metabolism than counterparts from healthy controls. However, further functional or metabolic studies are needed to confirm this connection.

*BCAR1* and *SKIDA1* were identified as differentially expressed in our study, although the absolute expression of these were below 1 TPM. Read distributions were consistently mapped to exons for *BCAR1* however, which may indicate that while low, the expression is reliable. *SKIDA1* on the other hand had no consistent read distributions between samples and few reads conforming to exons suggesting that this may be a spurious finding and its biologically relevance questionable. BCAR1 belongs to the CAS (Crk-associated substrate) adaptor protein family and is involved in Rap1 and chemokine signaling pathways. The protein is expressed in nearly all kinds of cells and serves as an important mediator for several infectious diseases although not much is known about its role in tolerance and autoimmunity (59). TLN2, being downregulated in APS-1s’ Tregs, is also involved in Rap1 signaling, but little is known about the biological function of this protein. Its paralogue, TLN1, encodes Talin-1 which is a cytoskeletal protein essential in mediating integrin activation. Talin-1 has been reported to have a role in Treg cell–mediated maintenance of immune homeostasis since T cell–specific deletion of Talin-1 in mice yielded lymphocyte activation due to Tregs deficiency (60, 61).

When only considering patients with the common 13bp deletion in exon 8 of *AIRE*, the genes *TMEM39B, AKR1C, GPSM3* and several members of the *HLA-DR/DQ* gene family emerged as differentially expressed compared to healthy controls. While it reduces the power of the differential expression analysis to include only a sub-cohort of patients, this is the largest subgroup in this study (N=5/9), and the 13bp deletion is also the most common Norwegian APS-1 mutation (20, 62). Although driven by a subgroup of healthy controls with high *HLA-DR/DQ*-expression, significantly lower levels of these genes were obvious in our analysis in patients with the 13bp *AIRE* mutation. HLA-DR/DQ-molecules are often expressed at high levels in Tregs and are considered as general activation markers for T cells. In fact, human CD4^+^CD25^hi^ T cells expressing HLA-DR/DQ have long been recognized as a functionally distinct population of Tregs that induces early contact-dependent suppression associated with high FOXP3 expression (63). Low levels of these could potentially indicate that the patients’ Tregs are less active than healthy controls, but functional studies are needed to confirm these findings. In addition to *TMEM39B*, the two final genes that were differentially expressed between APS-1 patients with the 13bp *AIRE* deletion and controls were *GPSM3* and *AKR1C3*. AKR1C3, also known as 17β-hydroxysteroid dehydrogenase type 5 is a steroidogenic enzyme (17β-HSD5) capable of converting prostaglandins to metabolizing estrogen and progesterone. GPSM3 in turn has a selective immune cell expression distribution and SNPs in this gene have been associated with autoimmune diseases. GPSM3 is upregulated when the level of myeloid-derived suppressor cells (MDSCs) expands, which again is correlated to increased levels of autoimmunity or inflammation. These MDSCs can also promote Treg development (64).

For our transcriptomic analyses, we have isolated Tregs based on the markers CD4^+^CD25^hi^CD127^-^ which should correspond to CD4^+^CD25^hi^FOXP3^+^ (65). Notably, even though APS-1 patients have fewer Tregs than control subjects in their blood, we have here included equal cell counts for subsequent analysis. Hence, it is not surprising that the classic five Tregs-markers were similar between patients and controls in our study. It has been previously reported that *FOXP3* expression levels are reduced in Tregs from APS-1 patients compared to healthy controls (16, 17), however we did not find a significant difference in *FOXP3* expression between patients and controls in this study. This may be explained by the lack of power due to a low number of patients included here or a selection bias as CD127^-^, which is correlated to FOXP3+ expression, was part of the cell sorting criteria for the RNAseq analysis. Nevertheless, sorting on CD4^+^D25^hi^CD127^-^ cells defines the nTregs coming from the thymus where AIRE has its most profound role, which is the reason why we chose to study this cell cohort. We do recognize that this leads to neglection of other blood borne suppressor cells which might have notable contributions to the total T suppressor cell pool as even patients with IPEX (hallmarked by FOXP3 deleterious mutations) have heterogeneous Treg-like cells in their blood (66).

In conclusion, we report here the first detailed transcriptomic analysis of Tregs from APS-1 patients. Although differences between Tregs of APS-1 patients and healthy controls were relatively subtle, we believe our findings provide important and additional clues to the dysfunctional Treg responses previously reported for APS-1 patients. Specifically, our findings implicate deficient gut homing, increased autophagy and cellular turnover, and increased metabolic activity in Tregs of APS-1 patients compared to healthy controls. Additional functional or metabolic studies are however needed to confirm the consequences of these transcriptional dysfunctions.

## Data Availability Statement

The datasets presented in this article are not readily available because of restrictions related to ethical matters regarding sharing of human transcriptomic data. Requests to access the datasets should be directed to the corresponding author (Anette.boe@uib.no). Access to the dataset requires an ethical approval from the requesting party.

## Ethics Statement

The studies involving human participants were reviewed and approved by the Regional Ethics Committee of Western Norway (approval numbers 2009/2555 and 2018/1417). The patients/participants provided their written informed consent to participate in this study. Written informed consent was obtained from the individual(s) for the publication of any potentially identifiable images or data included in this article.

## Author Contributions

AB, EB, SJ, EH, BO, and AW conceived and designed the study. ØB and EH recruited patients. AB, EB, TS, MH, TT, ØB, SJ, EH, BO, and AW acquired, processed, analyzed, and interpreted the data. AB, EB, SJ, EH, and AW drafted the manuscript. All authors critically revised the manuscript for important intellectual content. All authors contributed to the article and approved the submitted version.

## Funding

KG Jebsen Center for Autoimmune Disorders, Western Norway Health Authorities, and the Novo Nordisk Foundation (grant NNF17OC0027492).

## Conflict of Interest

The authors declare that the research was conducted in the absence of any commercial or financial relationships that could be construed as a potential conflict of interest.

## Publisher’s Note

All claims expressed in this article are solely those of the authors and do not necessarily represent those of their affiliated organizations, or those of the publisher, the editors and the reviewers. Any product that may be evaluated in this article, or claim that may be made by its manufacturer, is not guaranteed or endorsed by the publisher.
