# Supplementary material for: Transcriptional Changes in Regulatory T Cells From Patients With Autoimmune Polyendocrine Syndrome Type 1 Suggest Functional Impairment of Lipid Metabolism and Gut Homing
Source: Front Immunol. 2021 Aug 30;12:722860. doi: 10.3389/fimmu.2021.722860 (PMC8435668; doi:10.3389/fimmu.2021.722860)
Supplement: Supplementary file 1 [file DataSheet_1.docx]

**Supplementary Figure 1**

**1a**

**
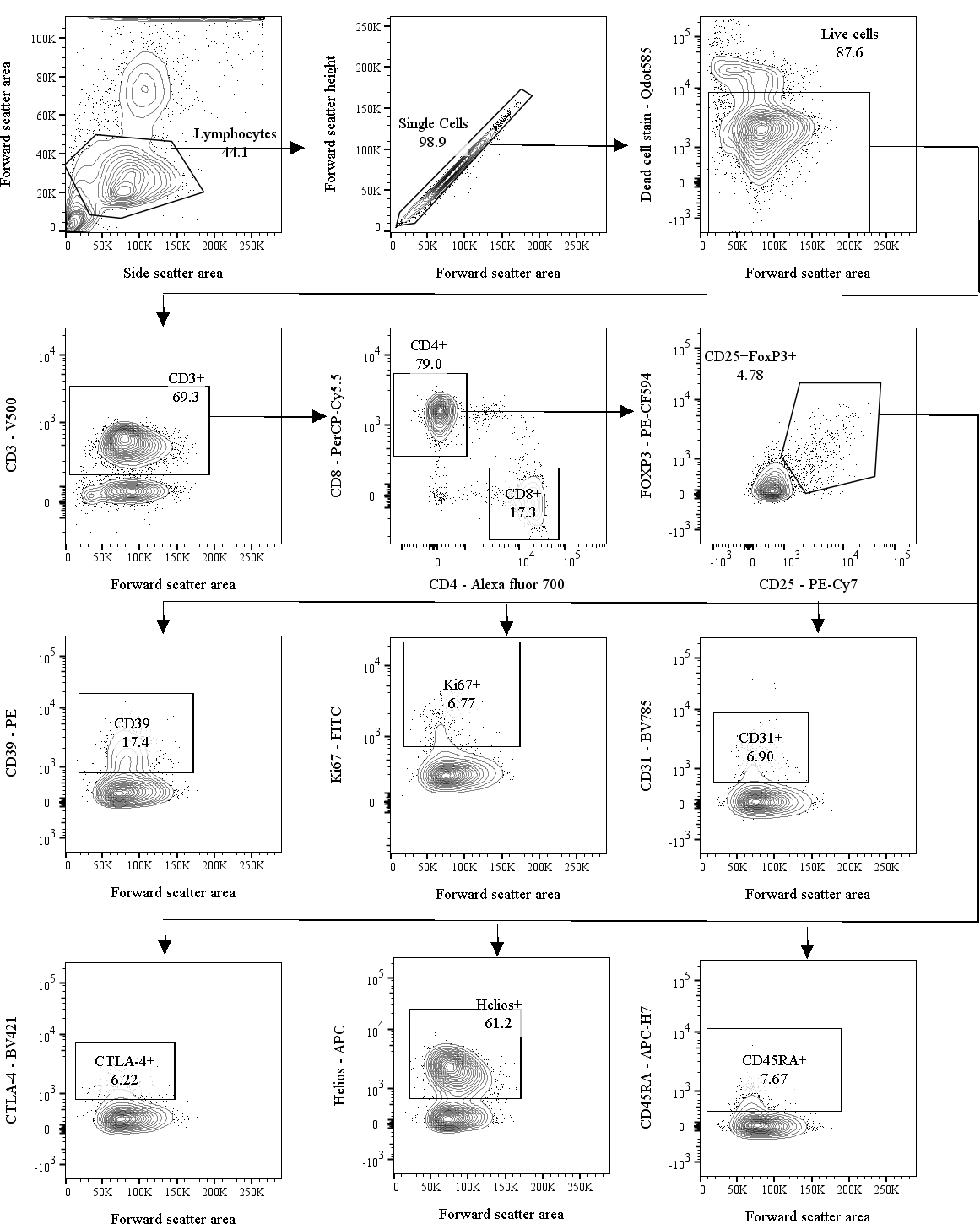
**

**1b**

**
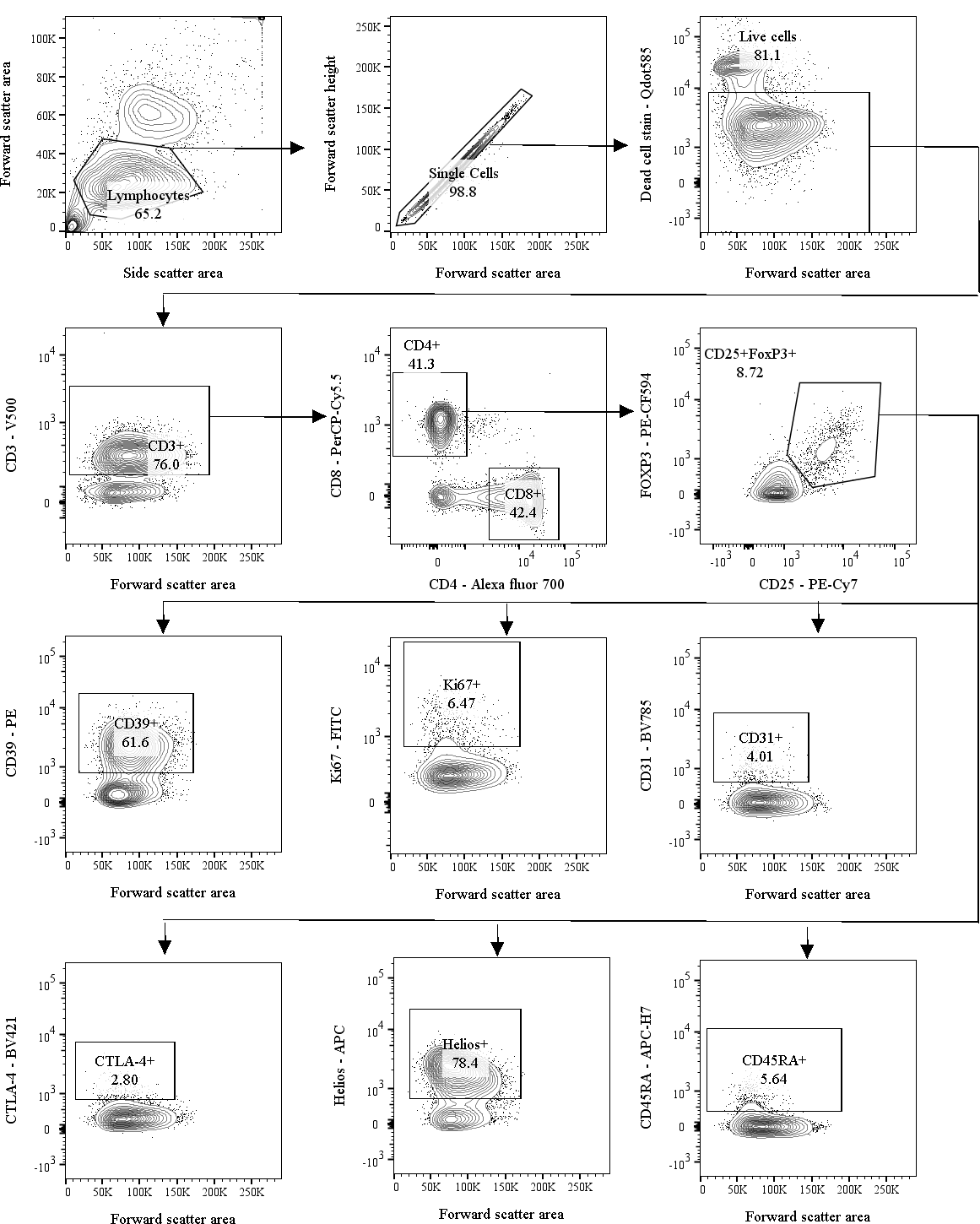
**

**1c**


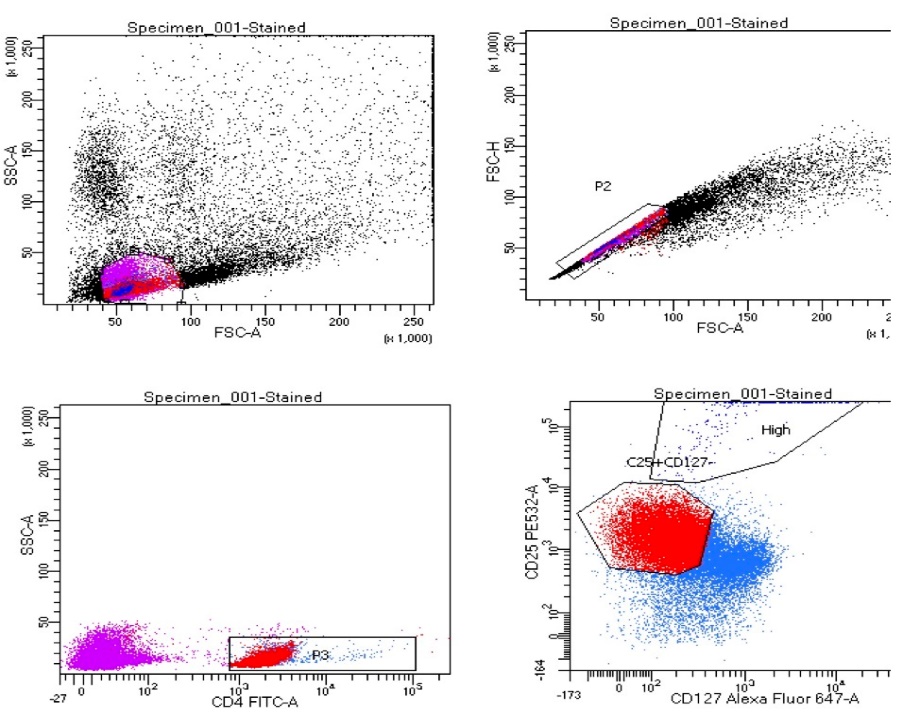

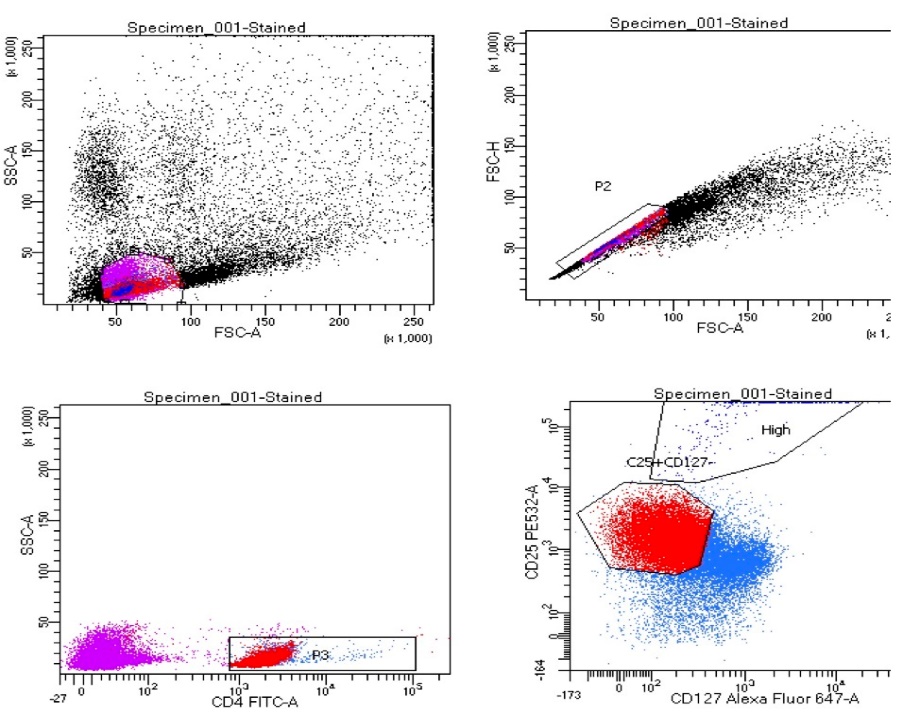


**Supplementary Figure 1** Gating strategies for **a.** The flow cytometry analysis of PBMC from a representative APS-I patient, **b**. The flow cytometry analysis of PBMC from a representative healthy control, and **c**. a representative patient in which magnetic pre-sorted Tregs are FACS sorted based on the CD4+ CD25+ CD127- phenotype. The antibodies that were used for these experiments can be seen from the x- and y-axes in the figure and are further described in the methodology section.


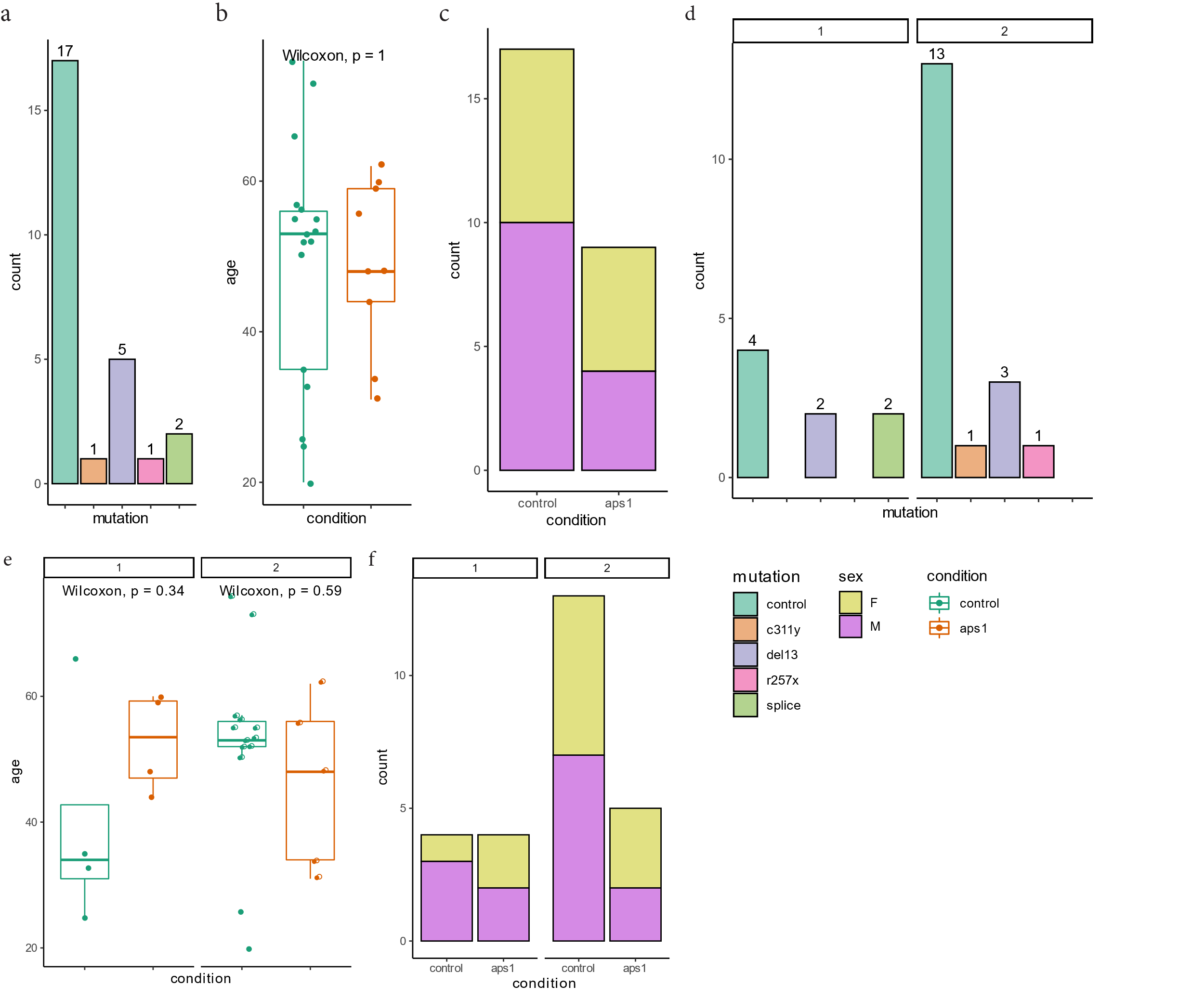
**Supplementary Figure 2**

**Supplementary Figure 2** Regulatory T-cells were isolated by FACS from 9 APS-1 patients and 17 heathy age and sex matched controls. The distribution of mutations in this study is shown in **a,** while **b** and **c** shows the age and sex distribution of patients and controls respectively. A Mann-Whitney *U* test was performed on the difference of age distribution in patients and controls and found no significant difference (p <0.05). The overview of the distribution of batches of patients and controls is shown in the histogram in **d** showing that the first batch was comprised of 4 controls, 2 patients with the 13bp-deletion mutation of AIRE and 2 patients with the splice mutation, while the second batch was comprised of 13 controls, 3 patients with the 13bp-deletion and one of each patient with the p.R257X and the p.C311Y mutations. **E** and **f** shows age and sex distributions of patients and controls respectively by batch. Mann-Whitney *U* test of age distribution between patients and controls within each batch show no significant difference (p <0.05)..


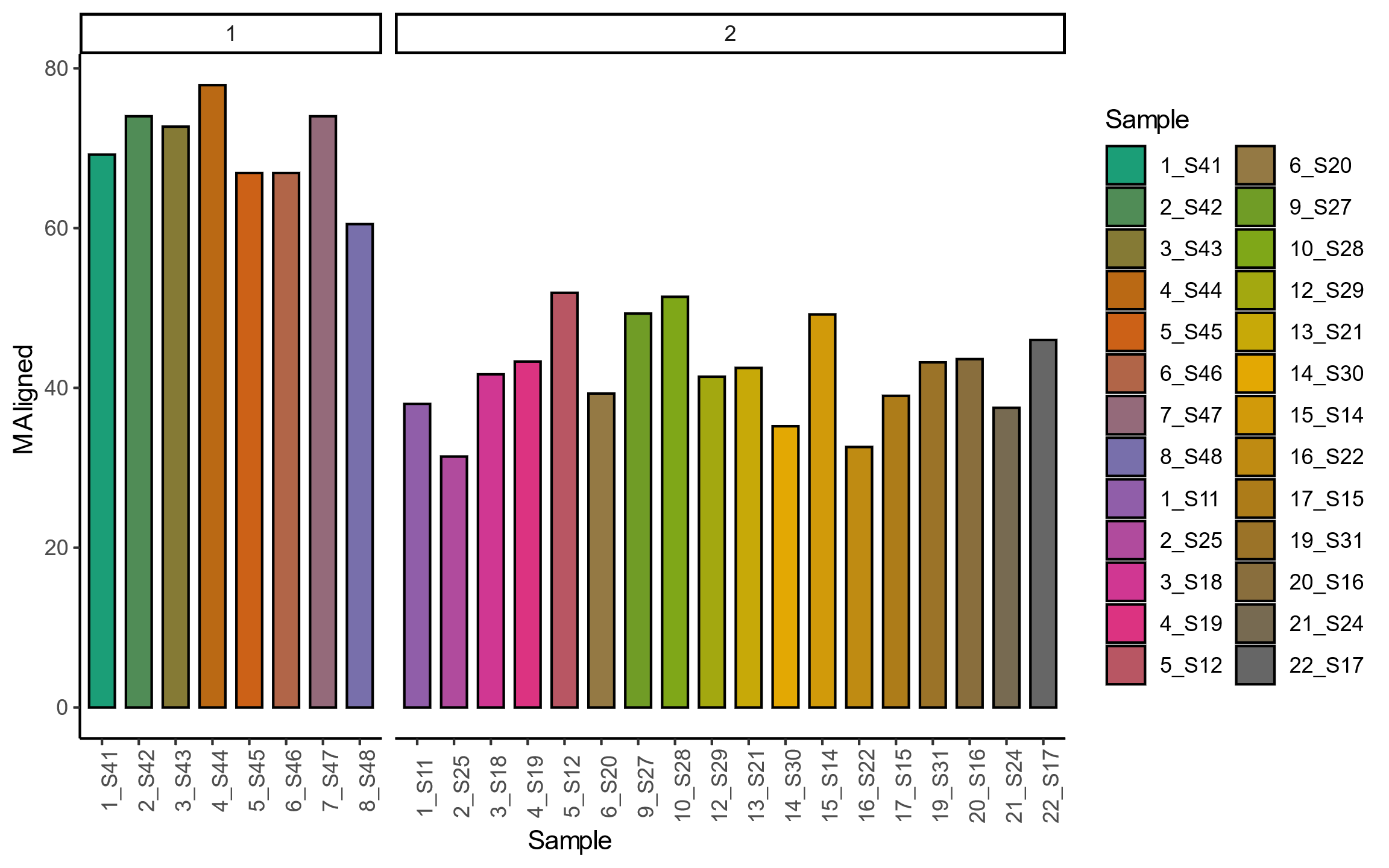
**Supplementary Figure 3**

**Supplementary Figure 3** Overview of sequencing depth of 5000 facs sorted regulatory T-cells from patients and controls in this study. Samples are grouped by sequencing batch.


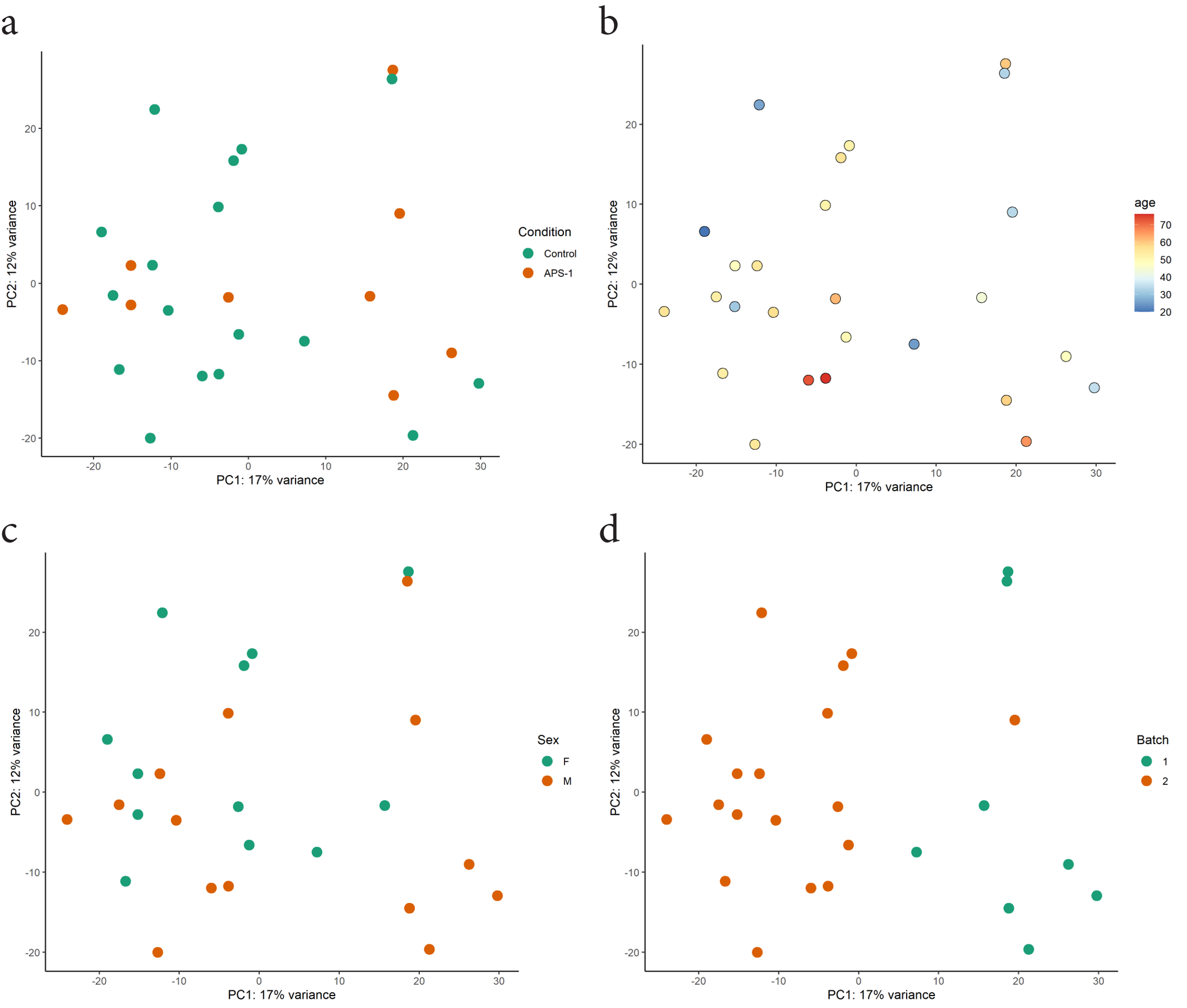
**Supplementary Figure 4**

**Supplementary Figure 4** PCA plots of the RNA sequencing data with the two principal components explaining the highest percentage of the variance in the data with PC1 explaining 17% of the variance and PC2 explaining 12% of the variance. **a** is coloured according to the condition of the samples, where green is control samples and orange is patients with APS-1; **b** is coloured according to age with the scale going from the youngest in blue to the oldest in red; **c** is coloured according to the sex of the participants, with green indicating females and orange indicating males; **d** is coloured according to which sequencing batch the samples are from with green indicating the first batch and orange indicating the second.


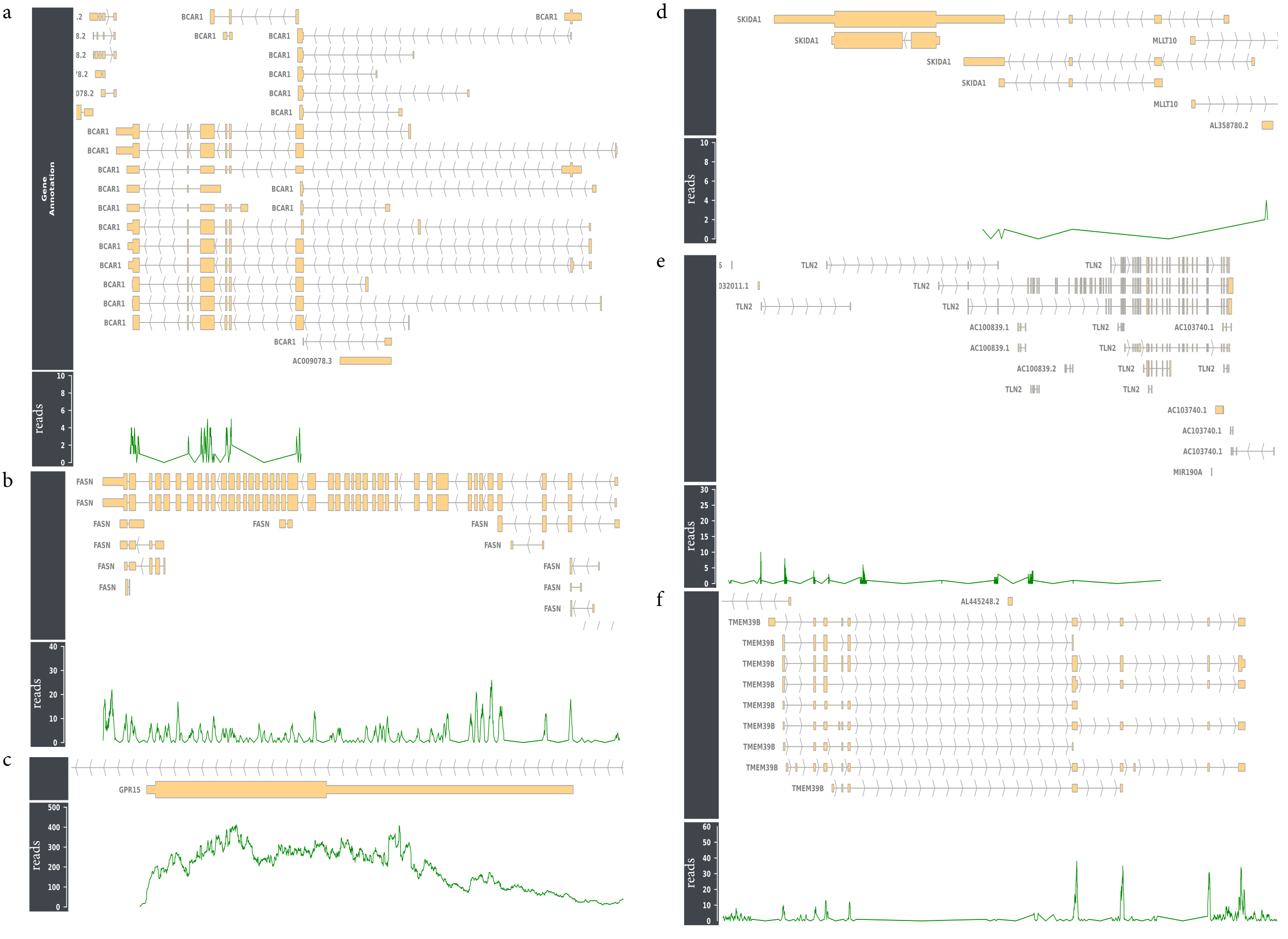
**Supplementary Figure 5**

**Supplementary Figure 5** Gene visualization using the Gviz r package of reads in genes found differentially expressed by DESeq2 with examples in selected control samples. BCAR1 in **a**, FASN in **b**, GPR15 in **c**, SKIDA1 in **d**, TLN2 in **e**, and TMEM39B in **f**.

**Supplementary Figure 6**

**
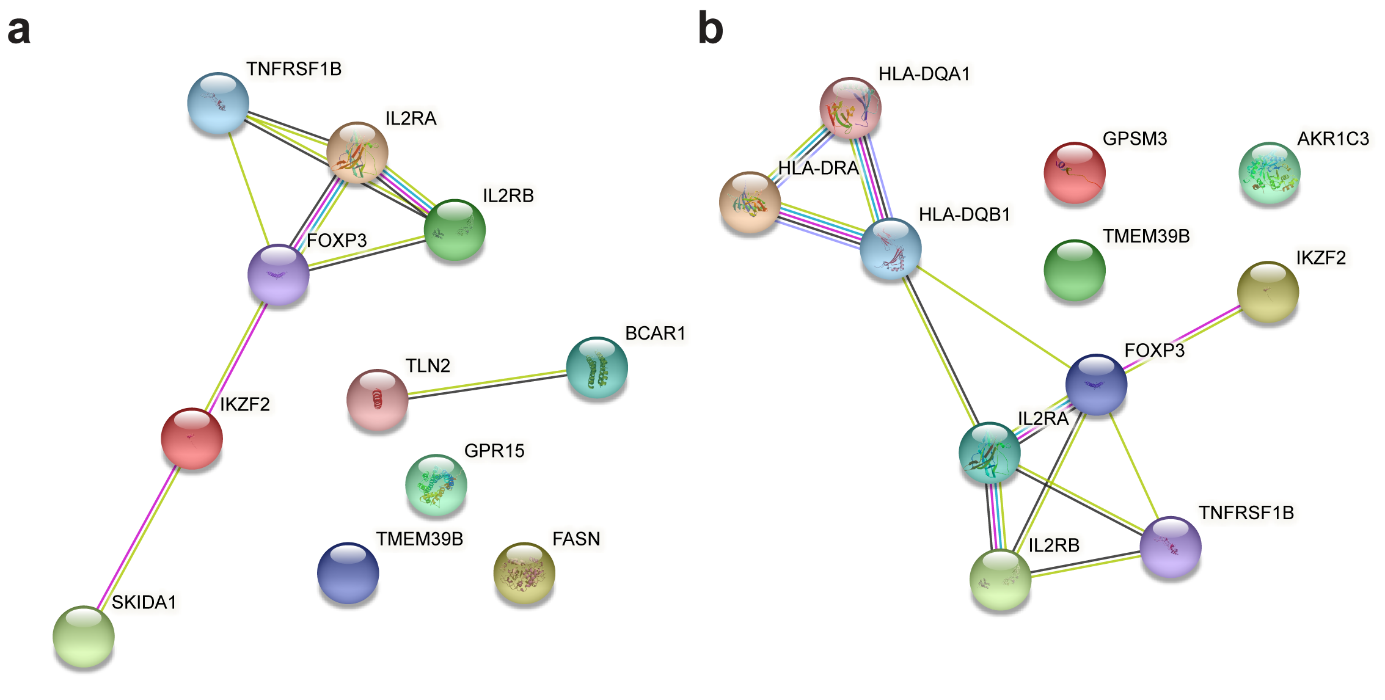
**

**Supplementary Figure 6 Network analysis of specific Tregs molecules (represented by FOXP3, ILZF2, IL2RA, IL2RB and TNFRSF1B) and significant up-/down- regulated genes or gene transcripts in APS-1 patients vs. controls from Table 1.** Network nodes represent proteins, edges represent protein-protein interactions (proteins which jointly contribute to a shared function). Known interactions from curative databases are dim blue in color while experimentally determined known interactions are pink. Predicted interactions are green (gene neighborhood), red (gene fusions) or dark blue (gene co-occurrence, while other interactions are yellow (textmining), black (co-expression) or purple (protein homology). **a)** Significant gene hits based on analyses of all APS-1 patients (N=9) vs controls (N=17). **b)** Significant gene hits based on analysis of APS-1 patients with the 13bp deletion in exon 8 of AIRE (N=5) vs controls (N=17).

**Supplementary Table 1: Pathway analysis of differential expressed genes and transcripts in Tregs from APS-1 patients and controls**

1. **Differential expressed genes. All patients (N=9) vs all controls (N=17)**

**From String-db.org**

|  |  |  |  |  |  |
| --- | --- | --- | --- | --- | --- |
| **Cellular components (all hits)** | **Description** | **Count in Network** | **Strength** | **FDR** | **Involved genes** |
| [GO:0001726](http://amigo.geneontology.org/amigo/term/GO:0001726) | Ruffle | 2 of 164 | 1.6 | 0.0420 | TLN2, BCAR1 |
|  |  |  |  |  |  |
| **KEGG pathways (all hits)** | **Description** | **Count in Network** | **Strength** | **FDR** | **Involved genes** |
| [hsa04510](https://www.kegg.jp/kegg-bin/show_pathway?hsa04510) | Focal adhesion | 2 of 197 | 1.52 | 0.0195 | TLN2, BCAR1 |
| [hsa04015](https://www.kegg.jp/kegg-bin/show_pathway?hsa04015) | Rap1 signaling pathway | 2 of 203 | 1.51 | 0.0195 | TLN2, BCAR1 |

1. **Differential expressed genes. Only 13bp del exon 8 patients (N=5) vs all controls (N=17)**

**From String-db.org**

| **Biological processes (all hits)** | **Description** | **Count in network** | **Strength** | **FDR** | **Involved genes** |
| --- | --- | --- | --- | --- | --- |
| [GO:0060333](http://amigo.geneontology.org/amigo/term/GO:0060333) | interferon-gamma-mediated signaling pathway | 2 of 69 | 2.05 | 0.0180 | HLA-DRA, HLA-DQ1 |
| [GO:0050852](http://amigo.geneontology.org/amigo/term/GO:0050852) | T cell receptor signaling pathway | 2 of 93 | 1.93 | 0.0180 | HLA-DRA, HLA-DQ1 |
| [GO:0019886](http://amigo.geneontology.org/amigo/term/GO:0019886) | antigen processing and presentation of exogenous peptide antigen via MHC class II | 2 of 96 | 1.91 | 0.0180 | HLA-DRA, HLA-DQ1 |
| [GO:0002684](http://amigo.geneontology.org/amigo/term/GO:0002684) | positive regulation of immune system process | 3 of 882 | 1.12 | 0.0180 | HLA-DRA, HLA-DQ1, GPSM3 |
|  |  |  |  |  |  |
| **Molecular function (all hits)** | **Description** | **Count in network** | **Strength** | **FDR** | **Involved genes** |
| [GO:0032395](http://amigo.geneontology.org/amigo/term/GO:0032395) | MHC class II receptor activity | 2 of 10 | 2.89 | 7.57e-05 | HLA-DRA, HLA-DQ1 |
| [GO:0042605](http://amigo.geneontology.org/amigo/term/GO:0042605) | peptide antigen binding | 2 of 22 | 2.55 | 0.00016 | HLA-DRA, HLA-DQ1 |
|  |  |  |  |  |  |
| **Cellular component (hits>strength 1.0)** | **Description** | **Count in**  **Network** | **Strength** | **FDR** | **Involved genes** |
| [GO:0042613](http://amigo.geneontology.org/amigo/term/GO:0042613) | MHC class II protein complex | 2 of 14 |  | 0.00048 | HLA-DRA, HLA-DQ1 |
| [GO:0071556](http://amigo.geneontology.org/amigo/term/GO:0071556) | integral component of lumenal side of endoplasmic reticulum membrane | 2 of 26 | 2.48 | 0.00055 | HLA-DRA, HLA-DQ1 |
| [GO:0030669](http://amigo.geneontology.org/amigo/term/GO:0030669) | clathrin-coated endocytic vesicle membrane | 2 of 35 | 2.35 | 0.00055 | HLA-DRA, HLA-DQ1 |
| [GO:0012507](http://amigo.geneontology.org/amigo/term/GO:0012507) | ER to Golgi transport vesicle membrane | 2 of 56 | 2.15 | 0.00085 | HLA-DRA, HLA-DQ1 |
| [GO:0032588](http://amigo.geneontology.org/amigo/term/GO:0032588) | trans-Golgi network membrane | 2 of 91 | 1.93 | 0.0017 | HLA-DRA, HLA-DQ1 |
| [GO:0005765](http://amigo.geneontology.org/amigo/term/GO:0005765) | lysosomal membrane | 2 of 252 | 1.49 | 0.0060 | HLA-DRA, HLA-DQ1 |
| [GO:0010008](http://amigo.geneontology.org/amigo/term/GO:0010008) | endosome membrane | 2 of 457 | 1.23 | 0.0134 | HLA-DRA, HLA-DQ1 |
|  |  |  |  |  |  |
| **Local network cluster (STRING) (all hits)** | **Description** | **Count in network** | **Strength** | **FDR** | **Involved genes** |
| [CL:18630](https://string-db.org/cgi/network?network_cluster_id=CL:18630&input_query_species=9606) | MHC class II protein complex | 2 of 10 | 2.89 | 6.89e-05 | HLA-DRA, HLA-DQ1 |
|  |  |  |  |  |  |
| **KEGG pathway (all hits)** | **Description** | **Count in network** | **Strength** | **FDR** | **Involved genes** |
| [hsa05310](https://www.kegg.jp/kegg-bin/show_pathway?hsa05310) | Asthma | 2 of 28 | 2.48 | 0.00061 | HLA-DRA, HLA-DQ1 |
| [hsa05330](https://www.kegg.jp/kegg-bin/show_pathway?hsa05330) | Allograft rejection | 2 of 35 | 2.35 | 0.00061 | HLA-DRA, HLA-DQ1 |
| [hsa05332](https://www.kegg.jp/kegg-bin/show_pathway?hsa05332) | Graft-versus-host disease | 2 of 36 | 2.34 | 0.00061 | HLA-DRA, HLA-DQ1 |
| [hsa04940](https://www.kegg.jp/kegg-bin/show_pathway?hsa04940) | Type I diabetes mellitus | 2 of 40 | 2.29 | 0.00061 | HLA-DRA, HLA-DQ1 |
| [hsa04672](https://www.kegg.jp/kegg-bin/show_pathway?hsa04672) | Intestinal immune network for IgA production | 2 of 44 | 2.25 | 0.00061 | HLA-DRA, HLA-DQ1 |
| [hsa05320](https://www.kegg.jp/kegg-bin/show_pathway?hsa05320) | Autoimmune thyroid disease | 2 of 49 | 2.2 | 0.00061 | HLA-DRA, HLA-DQ1 |
| [hsa05150](https://www.kegg.jp/kegg-bin/show_pathway?hsa05150) | Staphylococcus aureus infection | 2 of 51 | 2.19 | 0.00061 | HLA-DRA, HLA-DQ1 |
| [hsa05416](https://www.kegg.jp/kegg-bin/show_pathway?hsa05416) | Viral myocarditis | 2 of 56 | 2.15 | 0.00061 | HLA-DRA, HLA-DQ1 |
| [hsa05321](https://www.kegg.jp/kegg-bin/show_pathway?hsa05321) | Inflammatory bowel disease (IBD) | 2 of 62 | 2.10 | 0.00061 | HLA-DRA, HLA-DQ1 |
| [hsa04612](https://www.kegg.jp/kegg-bin/show_pathway?hsa04612) | Antigen processing and presentation | 2 of 66 | 2.07 | 0.00061 | HLA-DRA, HLA-DQ1 |
| [hsa05140](https://www.kegg.jp/kegg-bin/show_pathway?hsa05140) | Leishmaniasis | 2 of 70 | 2.05 | 0.00061 | HLA-DRA, HLA-DQ1 |
| [hsa05323](https://www.kegg.jp/kegg-bin/show_pathway?hsa05323) | Rheumatoid arthritis | 2 of 84 | 1.97 | 0.00061 | HLA-DRA, HLA-DQ1 |
| [hsa04658](https://www.kegg.jp/kegg-bin/show_pathway?hsa04658) | Th1 and Th2 cell differentiation | 2 of 88 | 1.95 | 0.00061 | HLA-DRA, HLA-DQ1 |
| [hsa05322](https://www.kegg.jp/kegg-bin/show_pathway?hsa05322) | Systemic lupus erythematosus | 2 of 94 | 1.92 | 0.00061 | HLA-DRA, HLA-DQ1 |
| [hsa04640](https://www.kegg.jp/kegg-bin/show_pathway?hsa04640) | Hematopoietic cell lineage | 2 of 94 | 1.92 | 0.00061 | HLA-DRA, HLA-DQ1 |
| [hsa04659](https://www.kegg.jp/kegg-bin/show_pathway?hsa04659) | Th17 cell differentiation | 2 of 102 | 1.88 | 0.00061 | HLA-DRA, HLA-DQ1 |
| [hsa05145](https://www.kegg.jp/kegg-bin/show_pathway?hsa05145) | Toxoplasmosis | 2 of 109 | 1.86 | 0.00061 | HLA-DRA, HLA-DQ1 |
| [hsa04514](https://www.kegg.jp/kegg-bin/show_pathway?hsa04514) | Cell adhesion molecules (CAMs) | 2 of 139 | 1.75 | 0.00076 | HLA-DRA, HLA-DQ1 |
| [hsa04145](https://www.kegg.jp/kegg-bin/show_pathway?hsa04145) | Phagosome | 2 of 145 | 1.73 | 0.00078 | HLA-DRA, HLA-DQ1 |
| [hsa05164](https://www.kegg.jp/kegg-bin/show_pathway?hsa05164) | Influenza A | 3 of 168 | 1.67 | 0.001 | HLA-DRA, HLA-DQ1 |
| [hsa05152](https://www.kegg.jp/kegg-bin/show_pathway?hsa05152) | Tuberculosis | 2 of 172 | 1.66 | 0.001 | HLA-DRA, HLA-DQ1 |
| [hsa05168](https://www.kegg.jp/kegg-bin/show_pathway?hsa05168) | Herpes simplex infection | 2 of 181 | 1.64 | 0.001 | HLA-DRA, HLA-DQ1 |
| [hsa05169](https://www.kegg.jp/kegg-bin/show_pathway?hsa05169) | Epstein-Barr virus infection | 2 of 194 | 1.61 | 0.0011 | HLA-DRA, HLA-DQ1 |
| [hsa05166](https://www.kegg.jp/kegg-bin/show_pathway?hsa05166) | HTLV-I infection | 2 of 250 | 1.5 | 0.0018 | HLA-DRA, HLA-DQ1 |
|  |  |  |  |  |  |
| **Annotated keywords (Uniprotein)** | **Description** | **Number** | **Count in network** | **FDR** | **Genes involved** |
| KW-0491 | MHC II | 2 of 14 | 2.75 | 0.00023 | HLA-DRA, HLA-DQ1 |
|  |  |  |  |  |  |
| **Protein domains (Pfam)** | **Description** | **Number** | **Count in network** | **FDR** | **Genes involved** |
| [PF00993 domains](https://pfam.xfam.org/family/PF00993) | Class II histocompatibility antigen, alpha domain | 2 of 6 | 3.12 | 1.02e-05 | HLA-DRA, HLA-DQ1 |
| [PF07654](https://pfam.xfam.org/family/PF07654) | Immunoglobulin C1-set domain | 2 of 24 | 2.24 | 0.00020 | HLA-DRA, HLA-DQ1 |
|  |  |  |  |  |  |
| **Protein domains and features (InterPro)** | **Description** | **Number** | **Count in network** | **FDR** | **Genes involved** |
| [IPR001003](https://www.ebi.ac.uk/interpro/entry/IPR001003) | MHC class II, alpha chain, N-terminal | 2 of 6 | 3.12 | 2.48e-05 | HLA-DRA, HLA-DQ1 |
| [IPR014745](https://www.ebi.ac.uk/interpro/entry/IPR014745) | MHC class II, alpha/beta chain, N-terminal | 2 of 15 | 2.72 | 6.03e-05 | HLA-DRA, HLA-DQ1 |
| [IPR003006](https://www.ebi.ac.uk/interpro/entry/IPR003006) | Immunoglobulin/major histocompatibility complex, conserved site | 2 of 38 | 2.31 | 0.00023 | HLA-DRA, HLA-DQ1 |
| [IPR011162](https://www.ebi.ac.uk/interpro/entry/IPR011162) | MHC classes I/II-like antigen recognition protein | 2 of 39 | 2.3 | 0.00023 | HLA-DRA, HLA-DQ1 |
| [IPR003597](https://www.ebi.ac.uk/interpro/entry/IPR003597) | Immunoglobulin C1-set | 2 of 43 | 2.26 | 0.00023 | HLA-DRA, HLA-DQ1 |
| [IPR007110](https://www.ebi.ac.uk/interpro/entry/IPR007110) | Immunoglobulin-like domain | 2 of 469 | 1.22 | 0.0156 | HLA-DRA, HLA-DQ1 |
| [IPR036179](https://www.ebi.ac.uk/interpro/entry/IPR036179) | Immunoglobulin-like domain superfamily | 2 of 478 | 1.21 | 0.0156 | HLA-DRA, HLA-DQ1 |
| [IPR013783](https://www.ebi.ac.uk/interpro/entry/IPR013783) | Immunoglobulin-like fold | 2 of 708 | 1.04 | 0.0260 | HLA-DRA, HLA-DQ1 |
|  |  |  |  |  |  |
| **Protein domains (SMART)** | **Description** | **Count in network** | **Strength** | **FDR** | **Genes involved** |
| [SM00920](http://smart.embl-heidelberg.de/smart/do_annotation.pl?DOMAIN=SM00920) | Class II histocompatibility antigen, alpha domain | 2 of 6 | 3.12 | 5.84e-06 | HLA-DRA, HLA-DQ1 |
| [SM00407](http://smart.embl-heidelberg.de/smart/do_annotation.pl?DOMAIN=SM00407) | Immunoglobulin C-Type | 2 of 39 | 2.3 | 8.53e-05 | HLA-DRA, HLA-DQ1 |
